# Supplementary material for: Local and Regional Dynamics of Native Maize Seed Lot Use by Small-Scale Producers and Their Impact on Transgene Presence in Three Mexican States
Source: Plants (Basel). 2023 Jun 30;12(13):2514. doi: 10.3390/plants12132514 (PMC10346707; doi:10.3390/plants12132514)
Supplement: Supplementary file 1 [file plants-12-02514-s001.zip › plants-2436645-supplementary.pdf]

5. Supplementary materials.

Table S1. Survey format. Comparison of seed management dynamics and cultural practices from producers of the three surveyed states. We grouped the responses according to the demographics of the maize producers and their production units; characteristics of their maize seed lots and exchange dynamics and agricultural practices.

Start date:	survey number:				
Locality/community/municipality:			ethnicity:		
Altitude:	Latitude:		Longitude:		
Survey questions	samples code				
	farmer-1	farmer-2	farmer-3	farmer-4	farmer-5
Name of producer:					
Producer's age:					
telephone number					

Indigenous language

collect name

Type of land ownership of property:

Ejido/communal/private

Num of types or varieties:

native, enhanced, hybrids

Planted area: Native, enhanced, hybrids

Name/color of maize sample collected

Main use of grain

-tortillas

-snacks

-atole

-drinks

-other

Cultivation system:

-monoculture

-associated

Seed origin:

-family

-self-sufficiency

-neighbors from other communities

-another community

-market

-Liconsa

-commercial/ agricultural firm

-other

Years of seed conservation

Where / in what do you keep the seed?

To whom have you donated/sold/lent seed?:

- Family

-neighbors

-neighbors from other communities

Level of technology:

-tractor

-machinery

-both	
**Use of**:	
-Fertilizers	
-Herbicides	
-Insecticides	

Table S2. Results from maize samples collected and analyzed for the presence of recombinant sequences by RT-PCR. The first screening for transgene presence was performed using probes for the P-35S and/or T-NOS sequences; samples positive for these sequences were further analyzed for three maize-specific events: NK603, MON810 and TC1507. Given the very low frequency of maize-specific events, here we grouped them together. For further details, see [47].

	**Mexico City**	**Chiapas**	**Oaxaca**
maize samples collected	263	472	656

maize samples analyzed for transgene presence	263	461	626
P35S	3	15	17
TNOS	2	15	36
positive	2%	13%	7%
negative	98%	87%	93%
percentage of samples positive for specific events	0	1.08%	1.27 %
